# The impact of memory support strategies on patient recall for treatment content in individuals with and without cognitive impairment

**DOI:** 10.21203/rs.3.rs-9152809/v1

**Published:** 2026-03-28

**Authors:** Anne E Milner, Crystal Woo, Linyan Ge, Sophia Oliver, Katrina Kuo, Kate Marcotullio, Joshua Varghese, Kiely Bol, Holly Bae, Sondra S Tiab, Laurel D Sarfan, Garret G Zieve, Joseph K Carpenter, Allison G Harvey

**Affiliations:** University of California, Berkeley; University of California, Berkeley; University of California, Berkeley; University of California, Berkeley; University of California, Berkeley; University of California, Berkeley; University of California, Berkeley; University of California, Berkeley; University of California, Berkeley; University at Albany, State University of New York; Department of Research and Evaluation, Kaiser Permanente Southern California; Oakland Cognitive Behavior Therapy Center; National Center for PTSD, Women’s Health Sciences Division; University of California, Berkeley

**Keywords:** Memory Support, Cognitive Impairment, Mild Cognitive Impairment, Sleep and Circadian, Transdiagnostic, TSC

## Abstract

The Memory Support Intervention was developed to improve poor memory for treatment by incorporating two different strategies into treatment: (1) constructive, where patients construct new ideas, inferences, or connections related to treatment content, and (2) non-constructive, which only highlight treatment content. The current study investigated the effects of delivering these strategies alongside treatment for midlife and older adults (≥ 60 years) with sleep difficulties, either experiencing cognitive impairment (CI; *n* = 29) or not (*n* = 30). Participants viewed video modules from the Transdiagnostic Intervention for Sleep and Circadian Dysfunction (TSC) and then received memory support. Aim 1 examined the effects of CI group and memory support type on recall. As expected, individuals with CI had poorer recall, but constructive memory supports reduced the difference between groups, suggesting constructive supports may be more effective than non-constructive memory supports in buffering against decline in recall for those with CI. Aim 2 evaluated the effects of CI and memory supports on thoughts and application of treatment content, but found no significant effects. Aim 3 examined how the number of treatment points affected recall. While an increase in points generally decreased recall, this was moderated by CI status and support type. Specifically, the decline in recall was most pronounced for the CI group following non-constructive supports. In contrast, constructive supports mitigated this impairment, resulting in comparable recall between groups when a larger amount of content was presented. Overall, constructive strategies may be particularly effective for helping individuals with CI better remember treatment content.

## Introduction

Patients often have poor memory for treatment content, accurately recalling only about one-third of treatment recommendations(e.g., Jansen et al., 2008; Lee & Harvey, 2015). Poor memory for treatment has been associated with poorer adherence(Dong et al., 2017; Kravitz et al., 1993; Linn et al., 2013) and poorer outcomes (Dong et al., 2017; Lee & Harvey, 2015; Sarfan et al., 2023). Midlife and older adults may be particularly vulnerable to poor memory for treatment, as memory function declines as we age (Salthouse, 2009), and older age is associated with worse recall of treatment content (Jansen, Butow, et al., 2008). Additionally, midlife and older adults commonly experience Cognitive Impairment (CI), which is broadly defined as a decline in cognitive functioning processes such as memory, learning, attention, and executive function (Morley, 2018). A type of CI, Mild Cognitive Impairment (MCI) is considered an intermediate stage between normal aging and potential progression to dementia (Petersen et al., 2001). Individuals with MCI experience more significant declines in cognition and memory functioning than what is typically observed during healthy aging (e.g., Kirova et al., 2015). As a result, individuals experiencing MCI may be further affected by poor memory for treatment compared to midlife and older adults who are aging without cognitive impairment.

MCI affects 12–18% of adults over the age of 60 (Alzheimer’s Association, 2022) and is a significant risk factor in progression to dementia. Specifically, 10–15% of individuals with MCI progress to dementia within one year (Petersen et al., 2001). This progression, coupled with the increased need for more frequent healthcare visits (Zhu et al., 2013), underscores the importance of interventions that support memory (e.g., for the content of healthcare visits) in older adults with MCI. Furthermore, individuals experiencing MCI face psychological distress if they are unable to recall information (Hurt et al., 2011). Importantly, past research conducted with people experiencing MCI indicates that interventions incorporating memory-based approaches have larger effect sizes on neuropsychological outcomes compared to multidomain approaches that target different cognitive domains and/or lifestyle factors (Sherman et al., 2017). This finding suggests that memory-focused interventions could be particularly effective for individuals experiencing MCI. However, it remains unclear which specific memory strategies are most effective for individuals with MCI in remembering treatment recommendations.

The Memory Support Intervention (MSI) (Harvey et al., 2014) was designed to improve memory for treatment with the goal of improving treatment outcome. The MSI is incorporated into treatment-as-usual. While psychosocial treatments often contain some level of memory support, the MSI incorporates a wider range of memory support strategies and at a higher frequency (Sarfan et al., 2023). The MSI consists of four *non-constructive* and four *constructive* memory supports (Zieve et al., 2019). *Constructive* memory supports (application, evaluation, categorization, and cue-based reminder) encourage patients to generate new ideas, inferences, and/or connections with treatment content (Chi & Wylie, 2014; Menekse et al., 2013). In contrast, *non-constructive* memory supports (attention recruitment, repetition, practice remembering, and praise recall) involve therapists highlighting treatment content and participants passively absorbing treatment content, rather than encouraging participants to generate ideas, inferences or connections. Zieve et al., (2019) demonstrated that constructive memory support strategies resulted in greater improvements in patient recall relative to non-constructive in a middle-aged sample without MCI (*M*_*age*_ = 43.41, *SD* = 10.27). However, it remains unclear if constructive or non-constructive memory supports may improve memory for content learned in treatment for individuals with MCI. As constructive strategies require higher cognitive engagement, individuals with MCI may have difficulty effectively using these strategies due to greater declines in cognitive functioning, including difficulties with constructive thinking (Matsuda & Saito, 2009). However, as previously highlighted, constructive strategies can be more effective than non-constructive and may still provide a greater benefit to those with MCI if they are able to effectively engage with the treatment content. The current study identified cognitive impairment (CI) using the Montreal Cognitive Assessment (MoCA; Nasreddine et al., 2005) with a cut-off score < 26 indicating MCI, rather than through gold-standard diagnostic procedures for MCI. Participants were categorized into a CI group (MoCA < 26) and a non-impaired control group (no-CI; MoCA ≥ 26). The study was designed to test whether these different types of memory supports would benefit midlife and older adults experiencing CI compared to those without CI, specifically investigating whether constructive memory support can help those with CI achieve similar treatment recall as non-CI individuals, mitigating CI-related deficits.

Given that memory has limited capacity (Miller, 1956), there may be a maximum amount of treatment content that can be presented before no further information can be retained i.e., a ceiling effect. As an exploratory analysis, we sought to examine whether the amount of treatment content delivered influences memory recall and whether different types of memory support would better support the presentation of a greater amount of information. This is an important question, as the amount of content delivered in a session can impact the amount of information retained, possibly providing valuable insights into the amount of information to present during a treatment session for both individuals with and without MCI.

The Transdiagnostic Intervention for Sleep and Circadian Dysfunction (TSC) (Harvey & Buysse, 2017) was chosen as the platform for implementing the MSI. TSC is a psychosocial treatment designed to target a range of sleep and circadian problems. Notably, sleep and circadian problems are prevalent among midlife and older adults (Fok et al., 2010; Foley et al., 1995; Neikrug & Ancoli-Israel, 2010). These problems are also highly relevant to individuals with MCI. Sleep disruptions are more frequent in individuals with MCI compared to sleep changes that occur during normal aging (Vaz Fragoso & Gill, 2007). Addressing sleep problems in individuals experiencing MCI is crucial as sleep disturbance in MCI is a potential risk factor in progression to dementia (Beaulieu-Bonneau & Hudon, 2009; Shi et al., 2018). Individuals with MCI, relative to healthy controls, also have greater wake after sleep onset, reduced total sleep time, lower sleep efficiency, and reduced time spent in rapid eye movement (REM) sleep (Beaulieu-Bonneau & Hudon, 2009; Maestri et al., 2015; Naismith et al., 2013). Research has shown that age moderated the effects of TSC on sleep disturbance such that increased age was associated with poorer response to TSC (Armstrong et al., 2022). Another study focused on the effects of TSC in midlife and older adults found that TSC, relative to usual-care, was associated with improvements in sleep disturbance, depression symptoms, and select sleep-wake parameters but not improvements for other important outcomes such as sleep-related and overall impairment (Sarfan et al., 2022). Thus, MSI strategies were integrated to test whether they improved memory recall for TSC content among midlife and older adults with and without MCI.

The present study was designed as an exploratory investigation to assess the comparative effectiveness of two types of memory support strategies, with the primary focus on the relative benefit of these strategies for individuals with CI compared to those without impairment. The study has three aims. Aim 1 was to evaluate whether (a) recall for treatment content was worse for individuals experiencing CI relative to those not experiencing CI and whether this effect was influenced by time since delivery of treatment content and (b) differences in memory recall for treatment content between the CI and no-CI group would be modulated by memory support type (constructive vs. non-constructive). We hypothesized that memory recall would be lower for those with CI than those without CI, the CI group would benefit more from constructive than non-constructive memory supports, and memory recall would be reduced after a 1-week delay for both groups. Aim 2 was to evaluate if thoughts and application of treatment content differed between CI and no-CI groups and if any differences were modulated by memory support type. We hypothesized that patients with CI would think less about treatment and implement fewer treatment points into their routine than those without CI. Aim 3 was to investigate the impact of the number of treatment points delivered during treatment on memory recall. We hypothesized that increased treatment content delivered would be associated with recalling proportionally fewer treatment points, and that the CI group would be more impaired by the delivery of a greater amount of treatment content than the no-CI group.

## Method

### Transparency and Openness

We report how we determined our sample size, and describe all data exclusions, manipulations, and measures in the study. All research materials, data, and analysis code are available from the authors upon request. The design and analysis of this study were not pre-registered, as this was an exploratory study and aimed to generate hypotheses for future confirmatory research.

### Participants

The University of California, Berkeley Committee for the Protection of Human Subjects approved the study (ID#: 2022–12-15881). Informed consent was obtained from all participants prior to the pre-treatment assessment. Participants (*N* = 59) were aged 60 and older (*M* = 70.60, *SD* = 6.86), 29 with CI and 30 without CI, based on cut-offs for defining mild cognitive impairment (described below). To reach a medium effect (Cohen’s *f* = .25) with α = .05 and power (1 - β) = .90 for a mixed design, a sample size of 46 was required. Thus, our sample size had sufficient statistical power. Participants were recruited within the United States through the distribution of flyers to senior centers, sleep clinics, churches, libraries, mental health clinics, memory centers, health professionals, and online forums. Participants were compensated $30 for each session, resulting in a maximum compensation of $90 for completing all three sessions. Data collection occurred from June 2023 to July 2024.

The inclusion criteria were: (1) aged 60 years or older, (2) exhibit a sleep or circadian disturbance defined as endorsing 4 (“quite a bit”) or 5 (“very much”), or the equivalent for reverse-scored items, on one or more PROMIS-SD questions (Yu et al., 2012), (3) English language fluency, (4) have a computer to use and stable internet connection, (5) able to attend sessions weekly and on the same day in sequential weeks i.e., 7 days apart. The exclusion criteria were: (1) The presence of substance abuse/dependence, mental illness, physical illness, suicidality, or neurological degenerative disease which would impact participation in the study or if there is a significant risk of harm and/or decompensation if treatment of that comorbid condition is delayed due to participating in this study, (2) Night shift work for more than two nights per week in the past 3 months (i.e., regularly scheduled work from 12 a.m. to 6 a.m.), (3) Not able and willing to participate in and/or complete the assessments.

### Measures

#### Memory Assessments

Memory was assessed using the Patient Treatment Recall Task and the Thoughts and Application Task.

The *Patient Treatment Recall Task* was used to assess participant memory for treatment contents (Lee & Harvey, 2015). It is a free recall task in which participants are asked to spend a minimum of 5 minutes to recall as many distinct treatment points as possible from the previous session. In the current study, treatment points were recalled verbally. The instructions were *“*Take a moment to think back to all the videos from our last session. Please say out loud as many distinct ‘treatment points’ as you can recall since the beginning of the last session. A ‘treatment point’ is knowledge or an insight, skill, or strategy that you think is important for you to remember and/or implement. You have 5 minutes for this task. Please take the entire 5 minutes so that we get every single point you remember.” A coding team consisting of a project coordinator and several undergraduate research assistants coded the recordings of participant recall task using a scoring rubric. This scoring rubric included each treatment point that was delivered during the treatment module videos (as determined by the research team). For each possible treatment point coders assigned 0 = treatment point not recalled and 1 = treatment point recalled. See Supplement Table 1 in the for a list of treatment points delivered in each module and Supplement Table 2 for a list of all memory supports used by type.

To further assess participant memory and learning, the *Thoughts and Application Task* was used, which comprises two separate sub-tasks: one to assess *thoughts* and one to assess *applications* (Gumport et al., 2015). The *Thoughts sub-task* assesses how often participants *thought* about therapy points learned from treatment in the past 24 hours. Participants are asked: “In the last 24 hours, have the contents of the videos from last week come to mind?”. If participants answer yes, they are asked “About how many times?”. Participants are then asked: “Within the past 24 hours, what came to mind?”. Similarly, in the *Applications sub-task* participants are asked if they *applied* any of the treatment points that they learned from the previous sessions in the past 24 hours: “In the past 24 hours, did you get to apply any of the contents covered in your sessions or use the skills you have been learning during the session?”, If participants answer yes, they are asked: “what did you apply?”. The overall number of treatment points from the thoughts and applications task were coded using the same manual as the *Patient Treatment Recall Task.* The number of treatment points from the Thoughts and Applications were summed separately for each task.

#### Acceptability

To assess the acceptability of the treatment videos and the memory supports, participants were asked to rate the following questions on a scale of 1 (‘not at all acceptable’) to 9 (‘very acceptable’). “How acceptable were the videos to you?” and “How acceptable were the questions I asked you after each video?”

#### Cognitive Impairment

The *Montreal Cognitive Assessment* (MoCA) was used to detect mild cognitive impairment during the eligibility screen (Nasreddine et al., 2005). Specifically, participants completed a variety of tasks that assessed attention, memory, executive functioning, language, visuoconstructional skills, and orientation. Participants were screened with either the full MoCA, the 5-minute MoCA, or the blind MoCA. Scores on tasks for each version of the MoCA were summed to produce a single score. For the full MoCA, scores range between 0 to 30, with scores 26 or above considered normal (Nasreddine et al., 2005). For the mini MoCA, scores range from 0 to 15, with scores above 12 considered normal (Nasreddine, 2021). The blind MoCA was administered to 2 participants who initially did not have access to Zoom, scores range from 0–22, with a scores above 19 considered normal (Pendlebury et al., 2013). Therefore, participants with scores above the threshold reported above were included in the no-CI group and those with a score below those reported above were included in the CI group. Participants were initially screened using the 5-minute MoCA to minimize participant burden. However, once data collection began, participant burden proved to be less of an issue than anticipated, so to provide a more comprehensive assessment, we transitioned to using the full MoCA.

#### Study Conditions

The sleep treatment content was administered through videos that were created by a licensed psychologist (AGH). Across sessions, participants viewed a total of 6 videos of treatment modules based on the Transdiagnostic Intervention for Sleep and Circadian Dysfunction (TSC; Harvey & Buysse, 2017). The videos varied in length, ranging from 4 to 7 minutes. The delivery of treatment via videos was chosen to ensure consistency of treatment point delivery across participants. The 6 treatment modules covered the following topics that were taken from TSC: Regular sleep-wake times, learning a wind-down routine, learning a wake-up routine, improving daytime functioning, stimulus control, and relax the mind strategies. The number of treatment points in each module varied from 4–7 points, and across the modules, a total of 33 treatment points were presented. There were eight repeated treatment points across modules. These repeated items introduced a methodological confound, as it was not possible to discern if memory for these items was learned in Session 1 or 2, where different memory supports were delivered. To eliminate this confound, repeated treatment points were not scored. This resulted in a total of 25 unique treatment points that were analyzed across sessions for each participant.

#### Sleep Coaches

Sessions were conducted by a sleep coach who presented participants with treatment modules and delivered constructive or non-constructive memory support. Sleep coaches underwent extensive training, which included directed readings, learning protocol scripts, viewing practice therapy sessions, learning treatment content from modules, learning memory support delivery techniques, and participating in mock sessions.

#### Memory supports

The memory supports used in the current study were from the Memory Support Intervention (MSI; Harvey et al., 2014). The MSI is comprised of eight different memory supports, distilled from the cognitive science and education literature. The MSI is designed to improve memory for treatment content with the aim of improving patient outcomes. Four of the memory supports are non-constructive and four are constructive.

#### Non-constructive memory support

The non-constructive memory supports that participants received during treatment were practice remembering, repetition, and attention recruitment. Praise recall was not included as a memory support in the current study due to difficulty in implementing this memory support in the study design. Participants received 6 non-constructive memory supports by sleep coaches after each video (18 total for the session). A double dose of non-constructive relative to constructive memory supports were used as previous data has shown that constructive memory supports are more effective than non-constructive at improving memory recall treatment content (Zieve et al., 2019).

#### Constructive memory support

The constructive memory supports used in the current study were application, cue-based reminder, and evaluation. Categorization was not included as a memory support as implementing this strategy was the most challenging and removing one of the memory supports resulted in having 3 non-constructive supports and 3 constructive supports to allow for equal counterbalancing of different types of memory supports to each participant. Participants received 3 constructive memory supports after each treatment module (9 total for the session). See Table 1 for examples of the constructive and non-constructive memory supports used in this study.

### Procedure

**Figure 1:**
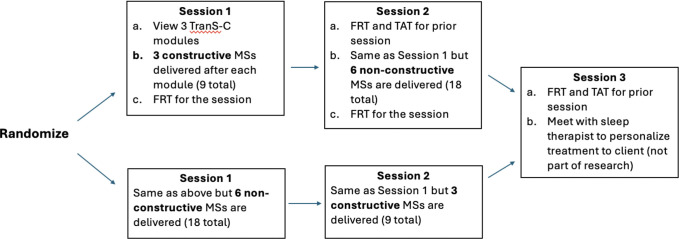
Participant flow for Sessions 1 – 3. *Note. FRT* = Patient treatment free recall task, *TAT* = Thoughts and Application Task.

For a diagram of the study flow please see Fig. 1. Potential participants were invited to an initial eligibility call over the phone. Eligible participants provided informed consent via DocuSign and completed a pre-treatment interview via Zoom consisting of a socio-demographic questionnaire. Participants then completed three 50-minute treatment sessions, each one week apart. Participants were randomized to either receive constructive or non-constructive memory support in Session 1. During Session 1, participants viewed 3 TSC treatment modules. The treatment modules delivered in each session where randomly selected from the 6 modules without replacement. After the delivery of each treatment module, a sleep coach delivered either constructive or non-constructive memory supports. At the end of Session 1 participants completed the Patient Treatment Recall Task for the session and rated the acceptability of the treatment videos and the memory supports. At the beginning of Session 2, participants completed the Patient Treatment Recall Task and Thoughts and Application Task for the prior session. Participants then viewed the remaining 3 TSC treatment modules that they had not viewed in the previous session, followed by the delivery of memory supports after each module. Participants who received constructive memory supports the prior week received non-constructive for Session 2 and vice versa for participants who received non-constructive the prior week (i.e., counterbalanced across participants). Participants then completed the Participant Treatment Recall Task for treatment content learned in Session 2. At the beginning of Session 3, participants completed the Participant Treatment Recall Task and Thoughts and Application Task for the previous session. No memory supports were delivered during this session.

### Analysis Plan

All data analyses were conducted in R (R Core Team, 2021). Data were manipulated using the dplyr package (Wickham et al., 2023) and analyzed using lme4 (Bates et al., 2015), emmeans (Lenth, 2024), and ggeffects packages (Lüdecke, 2018). Data were visualized using ggplot2 (Wickham et al., 2016). Due to experimenter error, the data for Session 1 for one participant from the no-CI group were not recorded. It was not possible to determine if the participant received the assigned conditions, so this participant was removed from the analyses. For each aim, multilevel modelling (MLM) was used to account for multiple observations nested within participant. After exclusion of this participant, there were no missing data. An alpha level of 0.05 was used. However, if the *p-*value was less than or equal to 0.10 but greater than 0.05, we discuss the results as marginally significant/trends. Across models all main effects and interactions are reported. Significant interactions were probed with pairwise comparisons and graphed. The Tukey method was used to adjust for multiple comparisons.

Aim 1 and 3 used multilevel logistic regression, as outcome of the Patient Treatment Recall Task as binary (i.e., 0 = treatment point not recalled and 1 = treatment point recalled). The estimates from the logistic regression model are expressed as proportions, representing the predicted probability of the outcome occurring at different levels of the predictors. To improve interpretability, we present these proportions as a percentage i.e., the percentage of recalled treatment points. Aim 1 evaluated the effects of CI group and memory support (MS) type on memory recall immediately post-session and after a 1-week delay (i.e., Patient Treatment Recall Task at the beginning of Session 2). The first level represents within-person variation and includes time indicators (0 = immediate, 1 = 1-week post session) and memory support type (0 = non-constructive, 1 = constructive) as predictors. The second level represents between-person variation in the intercepts and coefficients and includes CI group (0 = no-CI, 1 = CI) as a predictor. For Aim 1, the time-by-CI group, MS type-by-CI group, and time-by-MS type-by-CI group interactions were of primary interest. Aim 3 evaluated the effects of CI group, MS type, and number of treatment points delivered on memory recall. The first level represents within-person variation and includes memory support type (0 = non-constructive, 1 = constructive) as predictors. The second level represents between-person variation in the intercepts and coefficients and includes CI group (0 = no-CI, 1 = CI) and the continuous variable of number of treatment points delivered as predictors. For the latter, the effects are interpreted by comparing the percentage of treatment points recalled at the maximum and minimum number of treatment points that were delivered across sessions (range: 6–19). The number of treatment points delivered was mean centered and scaled to −1 to 1, where - 1 corresponds the minimum number of treatment points delivered (6) and 1 corresponds to the maximum number (19), as recommended in regression models to reduce multicollinearity (Cronbach, 1987). The model including all four predictors (Time, MS type, CI group, and number of treatment points delivered) failed to converge due to model complexities. Therefore, the models for Aims 1 and 3 were assessed separately such that each model included the primary interactions of interest and avoided including a four-way interaction which are difficult to interpret. The number of treatment points-by-CI group interaction and the three-way interaction of number of treatment points-by-CI group-by-MS type were of primary interest. Aim 2 used multilevel modeling to assess the effects of memory support type and CI group on the Thoughts and Application Task and acceptability ratings. The first level represents within-person variation and includes memory support type (0 = non-constructive, 1 = constructive) as predictors. The second level represents between-person variation in the intercepts and coefficients and includes CI group (0 = no-CI, 1 = CI) as a predictor. The MS type-by-CI group interactions were of primary interest.

## Results

Demographic variables for participants are presented in Table 2. As evident in Table 2, the CI and non-CI groups did not differ on any pre-treatment patient demographic variable except on race (*p* = 0.05), education (*p* = 0.01), and sleep-related impairment (*p* = 0.04). Specifically, for race, there were more participants who identified as being White in the non-CI group (93.33%) than in CI group (62.07%). For education, more participants had some college education, or completed college, or vocational school in the CI group (62.07%) than the non-CI group (33.33%). However, a greater number of participants had some graduate school education or had completed graduate school in the non-CI group (66.67%) than the CI group (27.59%). However, when examining education as a continuous variable (years of education), there was no significant difference between the two groups (*p* = 0.27). For sleep-related impairment, individuals experiencing CI had higher PROMIS-SRI scores relative to those not experiencing CI. To determine if any effects were due to differences in education level or sleep-related impairment, sensitivity analyses were conducted by rerunning the analyses with education or sleep-related impairment as a covariate. The results remained unchanged from those reported below. Overall acceptability of the memory supports was high (*M* = 8.30) and did not differ between memory support type or by MCI status (all *ps >* 0.05).

### Aim 1: Effects of time, CI group, and MS type on memory recall

See Table 3 for means and standard errors of estimated marginal means and Table 4 for multilevel modeling results examining the effects of time, MS type, and CI group on percent of memory recall from the recall task. There was a main effect of time (*b* = −1.02, *SE* = 0.16, *p* < 0.001), such that recall was significantly higher immediately following the session (56.11%) compared to one week after the session (38.78%). There was a main effect of MS type (*b* = −0.34, *SE* = 0.17, *p* = 0.04), such that memory recall was significantly higher following non-constructive memory support (48.94%) relative to constructive memory support (45.80%).

**Figure 2:**
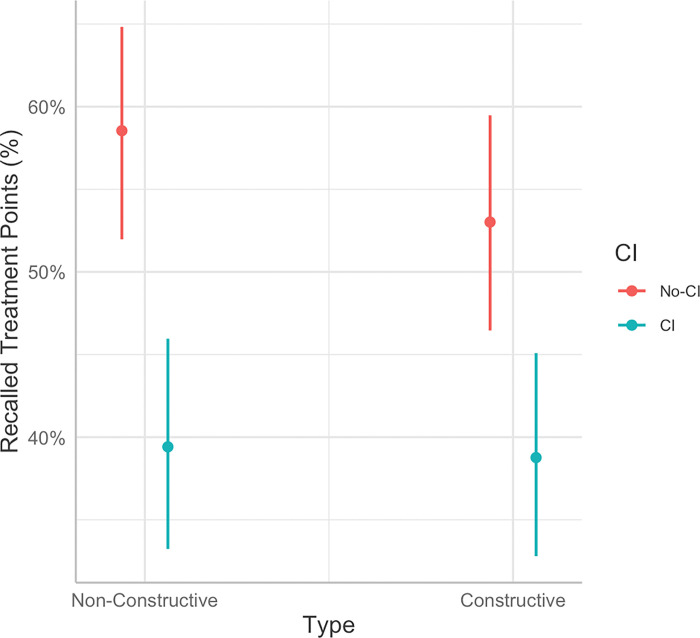
Interaction between CI group and memory support type (constructive vs. non-constructive) on the percent of recalled treatment points (%).

There was also a main effect of CI group (*b* = −1.07, *SE* = 0.23, *p* < 0.001), such that memory was significantly poorer in the CI group (39.09%) than the non-CI group (55.79%). However, there was a significant interaction between Time and CI group (*b* = 0.59, *SE* = 0.23, *p* = 0.01). Pairwise comparisons indicated that the effect of time on memory recall was significantly worse in the CI group relative to the no-CI group immediately following the session (*M*_*D*_^1^ = 21.42%, *p* < 0.001), but this difference was no longer significant one week later (*M*_*D*_ = 11.10%, *p* = 0.06). There was also an interaction between memory support type and CI group that approached significance (*b* = 0.37, *SE* = 0.23, *p* = 0.10; See Fig. 2). This interaction indicated that the effect of CI group on memory recall was such that the CI group had significantly poorer memory recall than the non-CI group following non-constructive memory supports (*M*_*D*_ = 19.13%, p < 0.001), but this difference was significantly smaller following constructive memory supports (*M*_*D*_ = 14.25%, *p* = 0.01). No other main effects or interactions were significant (*ps* > 0.10).

### Aim 2: Effects of CI group and MS type on thoughts and application

See Table 3 for the means and standard errors of estimated marginal means and Table 4 for the results of the multilevel models examining the effects of memory support type and CI group on thoughts and applications of treatment content from the thoughts and application task. There were no significant main effects or interaction between memory support type and CI group on the amount of treatment content that participants thought about in the past 24 hours (all *ps* > 0.10). Similarly, there were no significant main effects or interaction between memory support type and CI group on application of treatment content in the past 24 hours (all *ps* > 0.10).

### Aim 3: Effect of the number of treatment points delivered on memory recall

**Figure 3:**
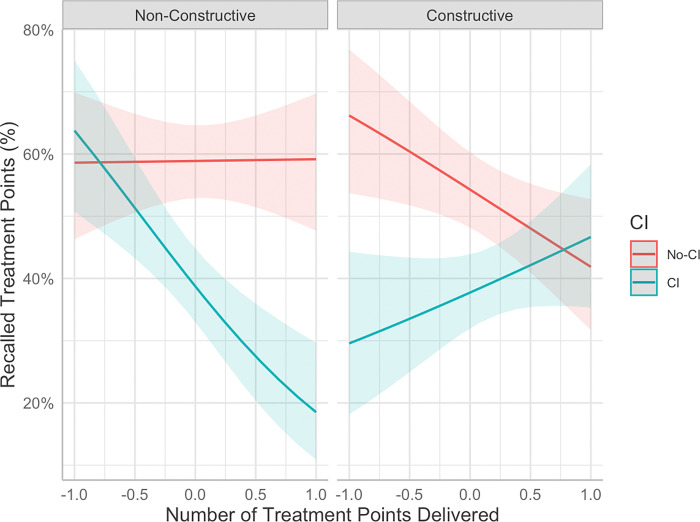
Interaction between CI group, number of treatment points delivered, and memory support type on the percent of recalled treatment points (%). *Note.* Number of treatment points was centered and scaled from −1 to 1. Raw number of treatment points delivered ranged from 6 to 19.

The average number of treatment points delivered per session was 13.51 (range: 6–19). See Table 4 for the results of the multilevel models examining the effects of number of treatment points delivered, MS type, and CI group on the percentage of treatment content recalled during the Participant Treatment Recall Task. Effects of MS Type and CI group, which were also reported in the Aim 1 model, followed a similar pattern previously reported. There was a significant interaction between CI group and number of treatment points delivered (*b* = −1.04, *SE* = 0.34, *p* = 0.002), such that the CI group relative to the non-CI group had significantly worse recall of treatment content when the maximum number of treatment points were delivered (*M*_*D*_ = 19.70%, *p* < 0.001) compared to when the minimum number of treatment points were delivered (*M*_*D*_ = 16.25%, *p* = 0.03). However, this interaction was qualified by three-way interaction between memory support type, CI group, and the number of treatment points delivered (*b* = 1.90, *SE* = 0.59, *p* = 0.001), such that the interaction between CI group and number of treatment points delivered differed by MS type (See Fig. 3). Following non-constructive memory supports, those in the CI group showed no differences from the non-CI group when a small number of treatment points were delivered (*M*_*D*_ = 5.16%, *p* = 0.56), but those in the CI group were significantly impaired relative to the non-CI group when presented with the maximum amount of treatment content (*M*_*D*_ = 40.66%, *p* < 0.001). In contrast, following constructive memory supports, participants in the CI group showed significant impairment in memory recall relative to those in the non-CI group when presented with less treatment content (*M*_*D*_ = 36.62%, *p* < 0.001), but not significantly different when presented with more treatment content (*M*_*D*_ = 4.79%, *p* = 0.56). In other words, the CI group performed no worse than the non-CI group when non-constructive memory support was delivered after a smaller number of treatment points or constructive strategies were delivered after a larger number of treatment points.

## Discussion

The goal of the present exploratory study was to test the impact of different types of memory support on memory recall and learning outcomes in individuals who are experiencing CI relative to midlife and older adults without CI. Specifically, this study evaluated whether *constructive* or *non-constructive* memory supports were more effective for those with CI relative to a control group without CI. We sought to evaluate whether the time since learning treatment content and the amount of treatment content delivered impacted memory recall.

The first aim was to evaluate (a) if recall for treatment differed between individuals experiencing CI and those not experiencing CI, and whether this effect was influenced by time since delivery of treatment content, and (b) if any differences between CI groups would be influenced by the type of memory support delivered. For both individuals with and without CI, memory recall was reduced one week after learning treatment content, suggesting that the retention of treatment content declines over time. This finding is consistent with classic research demonstrating that memory of learned information fades over time (Ebbinghaus, 1913). As expected, individuals experiencing CI had poorer recall for treatment content relative to those without CI, but only immediately following the session and not after a one-week delay, consistent with previous findings that individuals with CI have a reduced memory capacity (e.g., Kirova et al., 2015; Petersen et al., 1999). However, it is interesting that those with CI showed no deficit relative to those without CI after a one-week delay, suggesting that immediate recall was more greatly affected than long-term memory. The present study builds upon past work by demonstrating that individuals with CI are also impaired in retaining content learned in treatment. Across those with and without CI, memory recall was significantly improved following non-constructive memory supports, relative to constructive. However, this finding should be interpreted with caution due to the double dose of non-constructive relative to constructive memory support that may have resulted in this difference. As previously mentioned, the double dose of non-constructive memory support was delivered in an attempt to bolster the effects of non-constructive supports and constructive memory supports, given that constructive have been shown to be more effective (Zieve et al., 2019). However, the main effect of memory support type was qualified by a higher order interaction between memory support type and CI status, such that there was a smaller difference in memory recall between those with and without CI following constructive memory supports, relative to non-constructive, suggesting constructive memory supports may mitigate the negative effect of CI on recall, enabling those with reduced cognitive functioning to remember treatment content as well as those without cognitive impairment. This finding is particularly interesting, as even with the double dose of non-constructive memory support, these types of support were not as effective for those with CI. However, the interaction between CI group and both time and memory support type were marginally significant, tempering the strength of the conclusions that can be made, but given the small sample size, the current study may have been underpowered. Together, these results suggest that constructive memory supports are likely to better benefit individuals with CI than non-constructive memory support. These findings could be the result of constructive learning behaviors that encourage the learner to actively engage with the material by constructing new inferences, ideas, or connections and have been shown to improve learning outcomes (Chi et al., 2001) and subsequently improve memory (Zieve et al., 2019). By encouraging patient constructive learning behavior, these strategies may also help patients compensate for their impaired memory functioning. In contrast, non-constructive strategies that involve rehearsal-based strategies do not seem to offer the same benefits for individuals with CI. This finding aligns with research on interventions for the cognitive rehabilitation of memory to support individuals with CI, which typically distinguish between rehearsal-based approaches and compensatory approaches to improving memory (e.g., Hampstead et al., 2014; Simon et al., 2012). Rehearsal-based approaches share similarities with non-constructive strategies as they involve repetition of information over time, whereas compensatory strategies incorporate aspects similar to constructive learning behavior, such as using cues, organization of information, and elaboration, which aim to change how the patient learns, retains, or retrieves information. Such compensatory strategies likely promote constructive learning behaviors and allow patients to encode information more effectively, improving memory. Indeed, a meta-analysis found that compensatory strategies along with multidomain approaches were most effective for improving neuropsychological outcomes for individuals with CI, while rehearsal-based approaches were not associated with any improvement in outcome (Sherman et al., 2020). However, further research is needed to isolate the individual effects of compensatory interventions alone.

The second aim was to evaluate whether learning outcomes (i.e., thoughts and application of treatment content) differed between individuals with and without CI and by type of memory support. CI status and memory support type did not have a significant effect on the number of times participants thought about or applied treatment content in the previous 24 hours. It is possible that a larger sample may be needed to detect an effect with this task. Past research using this task has found significant effects when assessing the accuracy of thoughts and applications, with participants reporting more accurate thoughts and applications following the cognitive therapy (CT) plus memory support condition relative to CT-as-usual (Gumport et al., 2018). The treatment delivered in the current study used videos with treatment elements, which were less complex and covered a more limited range of treatment points compared to a typical TSC session. As a result, there were no inaccurate treatment points that were recalled in the current study across participants. Given these constraints, it is possible that this task yields minimal results when only examining the number of thoughts and applications reported by patients (i.e., the metric used in this study). However, assessing the accuracy of thoughts and applications could be more valuable in studies implementing more complex treatment sessions.

The third aim was to explore the impact of the number of treatment points delivered during treatment on memory recall. This is an important question as it provides valuable insights into the amount of information to present during a treatment session for both individuals with and without CI to optimize recall. Overall, it was found that as the number of treatment points delivered increased, the proportion of recalled treatment points decreased to a greater extent for those with CI relative to those without. This finding is consistent with past research that has reported that a higher cognitive load results in poorer memory performance (Sweller, 1994) with this effect being even more pronounced in older adults (Klencklen et al., 2017). Future research could establish an optimal number of treatment points to be delivered, which could help pinpoint the most effective amount of content healthcare providers should present to their patients. The effect of the number of treatment points delivered on memory recall differed by CI group and the type of memory support delivered. Specifically, following *non-constructive* memory supports, the effect was such that when fewer treatment points were delivered, there were no differences in recall between those with and without CI. However, as the number of treatment points delivered increased, memory recall for those with CI was significantly impaired relative to those without CI. In contrast, following *constructive memory* supports, those without CI demonstrated significantly improved recall relative to those experiencing CI when fewer treatment points were delivered. However, with an increasing number of treatment points delivered, this gap in performance reduced, and there were no significant differences between these two groups. This suggests that constructive memory supports may mitigate the effects of memory impairment when presented with a large amount of treatment content. These findings indicate that patients with CI should be presented with less treatment content to optimize memory, particularly when using non-constructive memory supports. However, the results also point to the possibility that constructive supports may be more effective in buffering against poor recall for those with CI when a large amount of treatment content is presented. Importantly, this finding highlights the importance of considering the number of treatment points presented to this population.

This study has several limitations. First, the videos of treatment elements were used to equalize the information available for encoding across participants. Thus, future research is needed to determine the generalizability of these findings to actual therapy sessions. Second, the use of a double dose of non-constructive memory supports meant that the effectiveness of constructive and non-constructive memory supports on memory could not be compared directly. Future research with the same dose across the two conditions is needed to make more concrete conclusions about the relative effectiveness of constructive versus non-constructive memory supports for individuals with CI. Third, while this study provides preliminary evidence for the comparative effectiveness of two types of memory supports, the lack of a control condition receiving no memory support limits our ability to isolate the magnitude of the MSI’s effect on recall. In future confirmatory research, we plan to employ a design with a control group that has not received memory support. Fourth, the current study was not designed to improve sleep per se, so further research is needed to determine if improvements in memory for the CI and non-CI groups resulted in improvements in sleep outcomes post-treatment. It is expected that improvements in memory for treatment would result in improved treatment outcomes, as has previously been demonstrated (Dong et al., 2017; Lee & Harvey, 2015; Sarfan et al., 2023). Fifth, the treatment sessions were conducted over Zoom, which provided some challenges to data collection, including technical difficulties such as poor internet connectivity and issues with screen sharing. Furthermore, conducting sessions remotely reduced the experimental control, as there were variations in the participants’ environments that may have affected consistent delivery of treatment content and measurement of memory tasks. Sixth, is that participants were screened using three different versions of the MoCA, which may have led to inconsistent screening across participants. However, all three versions of the MoCA used have been validated (Nasreddine, 2021; Nasreddine et al., 2005; Pendlebury et al., 2013). A final limitation of the study is that MoCA cut-off scores were used for screening instead of gold-standard diagnostic procedures for MCI. Consequently, the current research cannot be fully generalized to patients with a formal MCI diagnosis; they offer preliminary insights and provide a framework for future studies to directly test this population.

In conclusion, the present study demonstrated that there were no significant deficits in memory recall for the content of treatment among those with CI relative to those without CI following constructive memory support. However, those with CI had significantly impaired memory recall relative to those without CI following non-constructive memory support. These preliminary results suggest that constructive memory supports may be more effective than non-constructive for therapists to implement into treatment to enhance memory in individuals experiencing CI, particularly when patients are presented with a large amount of information during treatment. However, further work is needed to tease apart the effects of constructive and non-constructive memory support for individuals with CI. This study is a promising first step in demonstrating the possible benefits and acceptability of integrating memory support into treatment for individuals with CI.

## Figures and Tables

**Table 1:** Memory support strategies and examples

Strategy	Definition	Example
***Non-constructive Strategies***		
Practice remembering	Involves the therapist facilitating the patient to regenerate, restate, rephrase and/or revisit a treatment point	*“What are some of the main takeaways from this video?”*
Repetition	Involves the therapist restating, rephrasing, or revisiting a treatment point	*“To recap the main points in the video, you can improve your sleep health by avoiding taking naps during the day and not snoozing in front of TV at night”*
Attention recruitment	Involves the therapist using expressive language to direct attention to a treatment point	*“As you saw in the video, I can’t emphasize enough that it will make a HUGE difference if you wait until you feel sleepy before going to bed and avoid naps in the day if you can”*
**Constructive Strategies**		
Application	Involves the therapist working with the patient to apply a treatment point to the past, present or in the future	*“What might need to change in your life to apply the ideas from this video?”*
Cue-based reminder	Involves the therapist helping a patient develop a new or existing cue to facilitate memory for treatment points	*“What could we do to try to help you remember to think about and apply the content covered in this video this coming week?”*
Evaluation	Involves the therapist working with the patient evaluate (a) the advantages and disadvantages of a treatment point or (b) use comparisons to compare a new treatment point to an existing or hypothetical alternative	*“How would the new ideas discussed in the video compare to your current habits?”*

**Table 2:** Pre-Treatment Patient Demographics by CI group

Characteristic	No-CI (*n* = 30)		CI (*n* = 29)			
	*n*	%	*n*	%	χ^2^	*p*-value
Sex					0.14	0.71
Female	22	73.33	19	65.52		
Male	8	26.67	10	34.48		
Ethnicity					<0.01	1.00
Hispanic or Latino	1	3.33	0	0.00		
Not Hispanic or Latino	29	96.67	29	100.00		
Race					9.45	0.05
American Indian/Alaska Native	0	0.00	0	0.00		
Native Hawaiian/Pacific Islander	0	0.00	0	0.00		
Asian	2	6.67	5	17.24		
Black or African American	0	0.00	3	10.35		
White	28	93.33	18	62.07		
More than one race	0	0.00	1	3.45		
Other/category not listed	0	0.00	2	6.90		
Education					10.41	0.01
High school graduate or below	0	0.00	3	10.34		
Some or completed college or vocational school	10	33.33	18	62.07		
Some or completed graduate school	20	66.67	8	27.59		
Employment					1.33	0.72
Full-time	3	10.00	4	13.79		
Part-time	4	13.33	5	17.24		
Not employed	22	77.33	20	68.97		
Other/category not listed	1	3.33	0	0.00		
	**Mean**	**SD**	**Mean**	**SD**	**t**	**p-value**
Age	69.20	6.02	72.00	7.70	−1.53	0.13
Education (years)	17.60	2.37	16.70	3.81	1.13	0.27
PROMIS-SD	58.50	6.24	61.01	6.22	−1.55	0.13
PROMIS-SRI	53.23	9.86	58.01	6.99	−2.15	0.04
Full MoCA Score (*n* = 45)	28.14	1.25	23.04	1.85	10.89	<0.001
Mini MoCA Score (*n* = 12)	13.25	0.89	10.50	1.00	4.66	0.004
Blind MoCA Score (*n* = 2)	NA	NA	16.50	0.71	NA	NA

a Some patients endorsed more than one government assistance category *Note.* Chi-squared was used for categorical variables, and *t* tests were used for continuous variables. PROMIS-SD = PROMIS sleep disturbance; PROMIS-SRI = PROMIS sleep-related impairment.

**Table 3:** Estimated Marginal Means and Standard Errors from the Multilevel Logistic Regression Model (Aim 1, Expressed as Percentages) and the Multilevel Models (Aim 2).

	No-CI (n = 29)		CI (n = 29)	
	Mean	SE	Mean	SE
**Aim 1: Recall Task**				
*Post-Session*				
Non-constructive	70.18%	3.39%	45.86%	3.85%
Constructive	62.69%	3.65%	45.57%	3.74%
*Pre-Session (1-week later)*				
Non-constructive	45.86%	3.85%	34.39%	3.64%
Constructive	43.11%	3.79%	32.37%	3.39%
**Aim 2: Thoughts and Applications Task**				
*Thoughts*				
Non-constructive	2.36	0.39	2.18	0.39
Constructive	2.43	0.39	2.20	0.39
*Application*				
Non-constructive	2.30	0.36	2.35	0.36
Constructive	2.11	0.36	2.14	0.36

*Note.* MS = Memory Support, CI = Cognitive Impairment

**Table 4:** Coefficient Estimates from the Models Performed for Aims 1–3

Estimate	*b*	*SE*	*p*	*95% CI*
**Aim 1**				
Time	−1.02	0.16	**< 0.001**	[−1.35, −0.70]
MS Type	−0.34	0.17	**0.04**	[−0.67, −0.01]
CI group	−1.07	0.23	**< 0.001**	[−1.52, −0.63]
Time x MS Type	0.23	0.23	0.32	[−0.22, 0.67]
Time x CI group	0.59	0.23	**0.01**	[0.14, 1.05]
MS Type x CI group	0.37	0.23	0.10	[−0.08, 0.83]
Time x MS Type x CI group	−0.35	0.32	0.27	[−0.95, 0.25]
**Aim 2**				
*Thoughts*				
MS Type	0.06	0.42	0.15	[−0.75, 0.87]
CI group	−0.18	0.55	0.74	[−1.26, 0.89]
MS Type x CI group	−0.05	0.59	0.94	[−1.19, 1.10]
*Applications*				
MS Type	−0.19	0.29	0.52	[−0.76, 0.38]
CI group	0.05	0.51	0.93	[−0.95, 1.04]
MS Type x CI group	−0.02	0.42	0.96	[−0.83, 0.79]
**Aim 3**				
MS Type	−0.19	0.12	0.11	[−0.41, 0.04]
CI group	−0.82	0.18	**< 0.001**	[−1.17, −0.47]
Number of Treatment Points	0.01	0.21	0.96	[−0.41, 0.43]
MS Type x CI group	0.15	0.17	0.38	[−0.18, 0.47]
MS Type x Number of Treatment Points	−0.51	0.37	0.17	[−1.25, 0.23]
CI group x Number of Treatment Points	−1.04	0.34	**0.002**	[−1.71, −0.37]
MS Type x CI group x Number of Treatment Points	1.90	0.59	**0.001**	[0.73, 3.08]

*Note.* MS = Memory Support, CI = Cognitive Impairment, Bold indicates significant *p*-values

## Data Availability

The data that support the findings of this study are available from the corresponding author, AH, upon reasonable request.

## Footnotes

1. M_D_ = Mean difference
